# CFSH: Factorizing sequential and historical purchase data for basket recommendation

**DOI:** 10.1371/journal.pone.0203191

**Published:** 2018-10-10

**Authors:** Pengfei Wang, Jiansheng Chen, Shaozhang Niu

**Affiliations:** 1 Computer Science, Beijing University of Posts and Telecommunications, Beijing, China; 2 Institute of Remote sensing and Digital Earth, Chinese Academy of Sciences, Beijing, China; Victoria University, AUSTRALIA

## Abstract

To predict what products customers will buy in next transaction is an important task. Existing work in next-basket prediction can be summarized into two paradigms. One is the item-centric paradigm, where sequential patterns are mined from customers’ transactional data and leveraged for prediction. However, these approaches usually suffer from the data sparseness problem. The other is the user-centric paradigm, where collaborative filtering techniques have been applied on customers’ historical data. However, these methods ignore the sequential behaviors of customers which is often crucial for next-basket prediction. In this paper, we introduce a hybrid method, namely the *C*o-*F*actorization model over *S*equential and *H*istorical purchase data (*CFSH* for short) for next-basket recommendation. Compared with existing methods, our approach conveys the following merits: 1) By mining global sequential patterns, we can avoid the sparseness problem in traditional item-centric methods; 2) By factorizing product-product and customer-product matrices simultaneously, we can fully exploit both sequential and historical behaviors to learn customer and product representations better; 3) By using a hybrid recommendation method, we can achieve better performance in next-basket prediction. Experimental results on three real-world purchase datasets demonstrated the effectiveness of our approach as compared with the state-of-the-art methods.

## Introduction

Market basket analysis aims to discover meaningful patterns from massive customers’ purchase data [1]. It helps retailers to analyze the selling trends, to optimize the deployment of goods, and to understand customers’ preferences. With the prevalence of mobile applications and online e-commerce systems, market basket analysis becomes even more important in stimulating the consumptions and enlarging the selling profits, by providing the key technologies for personalized next-basket recommendation.

Generally, existing approaches to next-basket recommendation can be summarized into two paradigms. One is the item-centric paradigm. The key idea in the paradigm is “customers bought one product is also likely to buy some other products”. This recommendation paradigm has been applied to many e-commerce services such as Amazon. A number of approaches have been proposed to mine the meaningful sequential patterns from customers’ transactional data [2–4], which are similar to that of mining the association rules in data mining. However, the rule-based methods suffer from the data sparseness problem: the number of mined sequential patterns are always limited and it is hard to generalize the patterns to new products and users in real recommendation tasks. Although factorization methods have been proposed and applied on sequential patterns [5], these methods rely on individual sequential patterns and the data sparseness problem is not fully addressed.

Another approach is the user-centric paradigm. The key idea is “one is likely to buy the products favored by similar customers”. Collaborative filtering techniques have been applied [6–10]. A typical way is to represent customers’ historical purchase behaviors as a customer-product matrix where each entry represents the co-occurrence of the corresponding of customer and product in historical transaction. Matrix factorization is applied to learn the low-dimensional representations for both customers and products. In the method, the transactional sequence information is lost in the customer-product matrix. Therefore, the recommendation is made based on customers’ general interests. It is hard to capture the sequential purchase behaviors of customers, which is often crucial for next-basket prediction.

In this paper, we propose our hybrid method for the next-basket recommendation, namely *C*o-*F*actorization model over *S*equential and *H*istorical purchase data (*CFSH* for short). Specifically, on one hand, sequential pattern mining methods are applied to the massive transactional data to obtain the purchase sequential patterns. The mined patterns are represented as a product-product matrix. On the other hand, a customer-product matrix is constructed for representing the customers’ historical purchase behaviors, as that of in the conventional user-centric methods. These two matrices are then simultaneously factorized to learn the low-dimensional representations of both customers and products. With the learned representations, the next-basket prediction overcomes the problems that the previous approaches suffer from.

Compared with existing next-basket recommendation methods, our approach has the following advantages:

- By mining and factorizing global sequential patterns, it avoid the sparseness problem in traditional item-centric methods, which rely on the limited number of individual sequential patterns;
- By factorizing the product-product and customer-product matrices simultaneously, our approach fully exploits both sequential and historical behaviors to learn better representations for both customers and products;
- By adopting a hybrid recommendation method, our approach enjoys the advantages from both the user-centric paradigm and the item-centric paradigm, and thus achieved better performances in real recommendation tasks.

We conducted experiments over three real world purchase data sets: two from retailers and one from the e-commerce. Compared with the state-of-the-art baseline methods including the methods based on sequential pattern mining and the methods based on collaborative filtering, our approach performed significantly better. The results demonstrated the effectiveness of our approach in real world next-basket recommendation task.

## Related work

In this section, we provide background for basket recommendation. Two widely used recommendation models in market basket analysis, namely item-centric model and user-centric model, are introduced.

### Item-centric model

Sequential patterns have been widely observed in customers’ purchase behaviors and they are greatly useful in basket analysis. For example, when customers bought cameras, they probably bought SD-cards for the cameras in the next transaction. As an item-centric model, sequence mining is originally introduced for market basket analysis where the temporal relations between retail transactions are mined. Sequence mining is then extended to many other complex domains such as telecommunication, network detection, etc [11]. It is a topic of data mining concerned with finding statistically relevant patterns between data where the values are delivered in a sequence. Therefore, given the criteria support and confidence [12], early work on sequence mining algorithms like *ApriorALL*, *GSP* and *SPADE* are designed for mining frequent sequence of products [13]. These works focus on involving temporal dynamics into recommendations and lots of interesting patterns have been discovered [2]. In the past decades, many shopping malls have adopted sequential pattern analysis to discover temporal associations across transactions [10]. All these models focus on the observed sequential patterns, thus face the problem of data sparseness.

Recently, another simple but popular way used to model sequential patterns is concerning Markov assumption on customers’ sequential behaviors. These works factorize product-product matrix based on the mined sequential patterns to describe product transition in customers’ purchase behaviors [14]. Much of work focuses on learning representations for customers and products. Wang et al. [15] concern both intra- and inter-association patterns, and design a generative topic model to describe patterns’ distribution on a n-dimensional shopping interest space. Christidis et al. [7] explore the use of probabilistic topic models on transaction product sets to learn representations of both customers and products. Chen et al. [16] uses an n-gram model to predict which music customer will listen next, and to construct a playlist automatically given a seed music. Koenigstein et al. [3] describes product-product co-occurrences based on Markov chains. In another recent paper, Rendle et al. [5] use customer-specific Markov chains to model customers’ selection of products (*FPMC* for short). This model faces the data sparsity problem when factorizing the personal sequential patterns. The model assumes that all sequential patterns have the same weight for prediction. No statistics is conducted to validate whether the patterns are from the frequent product set. It is hard to ensure that intrinsic patterns are discovered.

In summary, most of the research regarding to sequential pattern mining focus on customers’ temporal behaviors. The approach leads to the following two difficulties: 1) Sequential pattern mining methods face the data sparse problem, and can only make recommendations base on observed rules. The ability of making personalized recommendation is limited. 2) Models based on sequential patterns did not consider historical purchase data, which makes the recommendation results often inaccurate and biased. Previous work uses a fixed size of sliding window to preserve historical products and generate recommendations [17]. However, it still fails to capture customer‘s general interest to products well. How to model customers’ historical data is still a big challenge in the approach.

### User-centric model

User-centric model is another widely used technique in market-basket analysis. Collaborative filtering is often used in the method to analyze customers’ historical purchase data. It can be further categorized into the memory-based approach and the model-based approach [18]. The memory-based approach provides the recommendation by studying the similarities among customers or products [4]. As an example, in e-commerce, retailers recommend one product to a customer because similar customers also purchases the product. The model-based approach tries to find a low-rank approximation of the customer-product rating matrix, and uses the values in the approximated matrix to recommend products [19]. As an example of model-based approach, matrix factorization is gaining rising attention in both explicit and implicit feedback applications such as Netflix [20, 21]. Many studies have been conducted. Lee [9] designed a binary customer-product matrix, and viewed the prediction problem as a two-class classification problem. Rendle et al. [22] assume that customers prefer the bought products than the ignored ones. They constructed product pairs as the training data and optimize for getting a correctly ranked product list rather than for assigning ranking scores for single products.

In many cases, however, the customers’ purchasing behaviors evolve over time. It is not reasonable to simply utilize the collaborative filtering methods for personalized recommendation, as the products purchased at different time periods might be significantly different. For example, a customer is likely to purchase candle and cake around her birthday, while she may be interested in electronic products in other days. Traditional collaborative filtering algorithms which describing customers’ preference based on customers’ historical purchase data fail to capture the evolution of customers’ purchasing interests effectively.

In conclusion, collaborative filtering seldom concerns the influence of customer‘s temporal interests to customers’ next purchase behaviors, and thus may limit the accuracy of prediction.

## Our framework

In this paper, we propose to factorize both customers’ sequential and historical purchase data for next-basket recommendation. By adopting such a hybrid recommendation method, our approach enjoys the advantages of both item-centric and user-centric paradigms. In the same time, these two paradigms complement each other and can achieve better performances. In this section, we will present the proposed method, namely the *C*o-*F*actorization model over *S*equential and *H*istorical purchase data (*CFSH* for short) in detail.

Specifically, we will first describe how to factorize the two matrices which are constructed based on the sequential and historical data for next-basket recommendation, respectively. The hybrid model *CFSH* is then presented based on the above two recommendation paradigms. Finally, we present the optimization algorithm for the proposed hybrid recommendation model.

### Notations

Let *I* = {*i*_1_, *i*_2_, …, *i*_|*I*|_} denotes the set of products, where |*I*| denotes the total number of unique products. Let *U* = {*u*_1_, *u*_2_, …, *u*_|*U*|_} denotes the set of customers, where |*U*| denotes the number of unique users. In transactional data, we use $T^{m}=\left\{t_{1}^{m},t_{2}^{m},\ldots ,t_{|T^{m}|}^{m}\right\}$ to denote the transactions corresponding to customer *u*_*m*_, ordered by the transaction-time, where |*T*^*m*^| denotes the number of transactions associated with user *u*_*m*_. Moreover, $|t_{k}^{m}|$ denotes the number of products involved in the *k* − *th* transaction of customer *u*_*m*_. We use *r*_*m*,*n*_ ∈ *R* to denote the times the product *i*_*n*_ ∈ *I* was purchased by the customer *u*_*m*_ ∈ *U* in the customer’s transactional data.

### Factorizing sequential purchase data

The item-centric paradigm has been widely adopted in next-basket prediction. The key idea is “who bought one product is likely to buy another”. Thus, it is critical to find the correlated products in this paradigm. To achieve the purpose, many approaches have been proposed to mine meaningful sequential patterns from customers’ transactional data [2–4], in a way similar to association rule mining in data mining.

However, an obvious drawback of traditional sequential modeling methods is the data sparseness, i.e., we statistic personal sequential patterns on a retail dataset with the weight larger than 1, we found that every customer obtained only 9.4 patterns. In our work, we propose to mine the sequential patterns in a global way, and factorize the mined patterns to obtain low dimensional production representations for better personalized recommendation. Here we first give the definition of sequential patterns in transactional data.

**Definition 1 *Sequential Pattern*** *Given the transaction set* $T^{m}=\left\{t_{1}^{m},t_{2}^{m},\ldots ,t_{k}^{m}\right\}$ *of customer u*_*m*_, *Sequential Pattern is defined as a weighted pair of products* <*i*_*a*_, *i*_*b*_, *w*_*ab*_>, *where* $i_{a}\in t_{m}^{u}$, $i_{b}\in t_{n}^{u}$, *m* < *n*, *and w*_*ab*_ *denotes the support of sequential pattern i*_*a*_ ⇒ *i*_*b*_.

**Fig 1:**
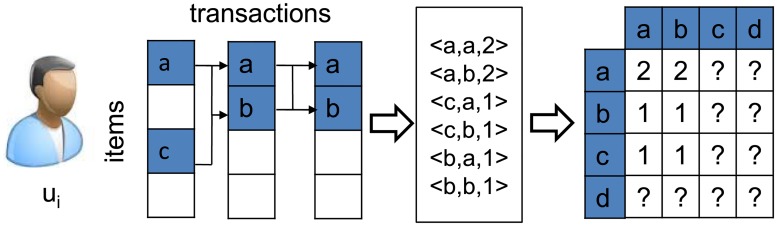
Contiguous patterns mined from a single customer, with quite a few patterns mined.

Existing work on sequential patterns focuses on the contiguous sequential pattern (*CSP* for short), by restricting the patterns mined from consecutive transactions of each individual customer [5, 12, 23]. Fig 1 shows an example. In this figure, a customer has three transactional records. Six *CSP*s are generated based on the records. It is obvious that the *CSP*s capture the local dependency of customer’s purchase behaviors. For example, a customer would probably buy a sim card in the next transaction if she bought a phone in the previous transaction. The mined *CSP*s can be represented with a matrix *W*, where element *w*_*a*,*b*_ corresponds to the pattern <*i*_*a*_, *i*_*b*_, *w*_*ab*_>.

**Fig 2:**
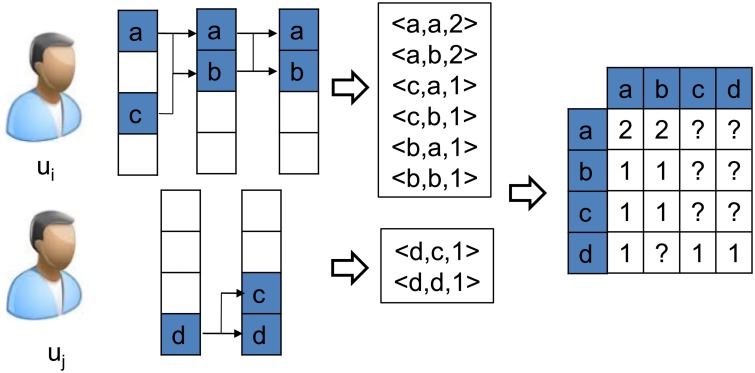
Global transition matrix gathered all customers’ sequential patterns. Element with? are missing values.

The *CSP*s mined from individual purchase data, however, are extremely sparse. Therefore, simply applying these patterns in recommendation systems would result in poor generalization performance. Researchers also proposed to collect the sequential patterns from all the users and further factorize these patterns to obtain low dimensional representations, as shown in Fig 2. In recent, Rendle et al. [5] proposed to assemble all the customers’ sequential pattern matrices into a tensor and then apply factorization techniques over the tensor. However, since the objective of the factorization is to recover the sparse local patterns in essence, it would be very sensitive to the noise in individual data.

To address the problem, we propose to assemble all the customers’ sequential pattern matrices into one matrix and apply factorization over this matrix. In this way, we can make the factorization focus on those globally salient patterns and more robust to the noise in the individual purchase data.

Specifically, we mine the *CSP*s from each customer’s transactional data, gather all the *CSP*s, and represent them into one global sequential pattern matrix *W*, with the element *w*_*a*, *b*_ corresponding to the pattern <*i*_*a*_, *i*_*b*_, *w*_*ab*_> where *w*_*ab*_ denote the overall weight of the pattern *i*_*a*_ ⇒ *i*_*b*_ in the transaction data. We then factorize *W* to learn the low dimensional representation of the products using the following objective function.

$$\begin{array}{c} \text{minimize}\{∥W-QQ^{T}{∥}^{2}+\lambda ∥Q{∥}^{2}\} \end{array}$$ (1)

where $Q\in R^{n\times k}$ denotes the k-dimension product representation matrix, and λ is the regularization coefficient.

Based on the learned low dimensional product representations, we can then provide personalized next-basket recommendation based on the customer’s latest transaction information. Specifically, we can inference the preference of customer *u*_*m*_ to product *i*_*n*_ in the next *k*-th transaction based on the products he bought in the latest transaction as follows:

$$\begin{array}{c} \begin{array}{c} pref_{m,n}=\sum_{l\in t_{k-1}^{m}}q_{n}*q_{l} \end{array} \end{array}$$ (2)

where *q*_*l*_ ∈ *Q* and *q*_*n*_ ∈ *Q* indicate representations of product *i*_*l*_ and *i*_*n*_ respectively,operator “*” means dot product of two vectors. With the customer’s score on each product, we can sort the products and obtain the top-*K* products to be recommended to customers.

**Fig 3:**
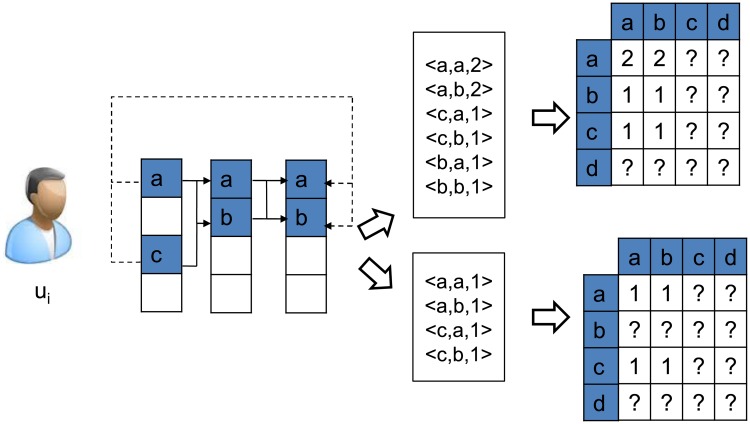
*CSP*s and *NCSP*s mined from transactions of a single user. Solid lines stand for *CSP*s, while dotted lines represent *NCSP*s with the skip of 1 mined from transactions of user *u*_*i*_.

In next-basket prediction, factoring contiguous sequential patterns makes the assumption of “customers’ next purchase behavior is only related to what he has bought in the most recent transaction”. That is to say, the model only takes the local dependency between adjacent transactions into consideration and ignores the long dependency ignored. However, we can imagine there actually are some long dependency among customers’ transactions. For example, when a customer purchased a phone, he may probably buy a phone case after using one month and thus these two purchase behaviors are no longer in two consecutive transactions. To further capture the long dependency between transactions, we can involve the non-contiguous sequential pattern (*NCSP* for short) in our sequential pattern mining procedure. Here non-contiguous sequential pattern refers to the sequential pattern mined from non-consecutive transactions, as shown in Fig 3.

There are two ways to leverage the *NCSP*s and *CSP*s in learning of the low dimensional product representations for recommendation. One simple way is merging these two sets of the patterns into one set. That is to combine all the *NCSP*s and *CSP*s to form the new sequential pattern matrix *W*, and apply the matrix factorization to learn the product representations. With the learned representations, we then obtain personalized recommendation for each customer based all his historical transactions.

The other way is to treat different sequential patterns differently. Specifically, we propose a line combination model over the *NCSP*s and *CSP*s.

$$\begin{array}{c} \begin{array}{cc} & \text{minimize}\{\sum_{i}a_{i}\sum ∥W_{i}-QQ^{T}{∥}^{2}+\lambda ∥Q{∥}^{2}\}\\ & \text{s}.\text{t}.\;\{\begin{array}{c} Q\geq 0\\ \sum \alpha_{i}=1 \end{array} \end{array} \end{array}$$

where *W*_*i*_ denotes the matrix of *NCSP*s with the skip of *i* transactions, and *α*_*i*_ represents the influence of each *NCSP*s to users’ purchasing behaviors. Obviously, *W*_0_ corresponds to the matrix of *CSP*s.

With the learned the low dimensional product representations, we can also use the linear combination model to provide personalized recommendation based on each customer’s historical transactions.

### Factorizing historical purchase data

As aforementioned, item-centric paradigm mainly leverages the correlation between products for recommendation. Another way is to exploit the correlation between users, so called user-centric paradigm. The idea behind is that “one is likely to buy the products favored by similar customers”, where collaborative filtering techniques have been widely applied. A typical way is to represent customers’ historical purchase behaviors as a customer-product matrix, and apply matrix factorization to learn the low-dimensional representations of both customers and products for recommendation.

**Fig 4:**
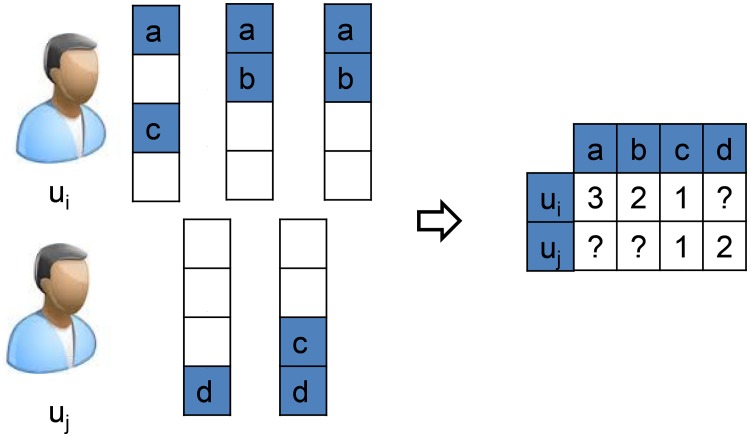
Customer-product matrix mined from transactions. Users’ purchase count to a certain product indicates customers’ general interest to it. The customer is more likely to buy a product in the next transaction the one he bought frequently before. Elements with? indicate unobserved purchase count.

Specifically, we first construct the customer-product matrix *R* based on customers’ historical transaction data, as shown in Fig 4:

We then factorize the customer-product matrix *R* to learn both the low dimensional representations of customers and products with the following objective function.

$$\begin{array}{c} \begin{array}{c} \text{minimize}\{∥R-PQ^{T}{∥}^{2}+{\lambda (∥P∥}^{2}+{∥Q∥}^{2})\} \end{array} \end{array}$$ (3)

where *u*_*m*_ is represented as a vector *p*_*m*_ in *P*, $P\in R^{m\times k}$. We assume *P* indicating customers’ static preference to products. The task of this model is to learn customers’ general tastes to products.

Based on the learned representations of customers and products, we can provide personalized next-basket recommendation by calculating the customer’s preference on each product as follows:

$$\begin{array}{c} \begin{array}{c} pref_{m,n}=p_{m}*q_{n} \end{array} \end{array}$$ (4)

where *p*_*m*_ and *q*_*n*_ are representations of user *u*_*m*_ and product *i*_*n*_ respectively. We then sort all products according Eq (4) and recommend top K products to each user.

### Hybrid method

The two recommendation paradigms described above provide personalized next-basket prediction from different ways, i.e., one leverages the correlation between products and the other relies on the correlation between customers. The previous one can well capture the sequential behaviors of customers and the latter can better model customer’s general interests by ignoring transactions and assuming the exchangeability of purchased products. Therefore, a natural idea is to combine the two recommendation paradigms so that we can enjoy the powers of both paradigms and meanwhile complement each other to achieve better performance.

In our hybrid recommendation method, we propose to simultaneously factorize the product-product matrix and user-product matrix. In order the make the alignment between the learned representations for products, we require that they share the same low dimensional representations of products. Therefore, the objective function is described as follows:

$$\begin{array}{c} \begin{array}{cc} & \text{minimize}\{\alpha ∥R-PQ^{T}{∥}^{2}+\beta ∥W-QQ^{T}{∥}^{2}+{\lambda (∥P∥}^{2}+{∥Q∥}^{2})\}\\ & \text{s}.\text{t}.\{\begin{array}{c} P\geq 0\\ Q\geq 0\\ \alpha \geq 0,\beta \geq 0,\alpha +\beta =1 \end{array} \end{array} \end{array}$$ (5)

Where *α*, *β* denotes the influence of users’ general tastes and temporal tastes to the next purchase behaviors. When *β* = 1, this model reduces to factoring customers’ sequential patterns, when we set *α* = 1, our model concerns only customers’ historical purchase behaviors.

With the learned low dimensional representations of customers and products, we can provide personalized next-basket recommendation with the same linear model using customers’ general interests as well as the latest transaction. Specifically, the preference of customer *u*_*m*_ to the product *i*_*n*_ can be inferenced as follows:

$$\begin{array}{c} \begin{array}{c} pref_{m,n}=\alpha p_{m}*q_{n}+\frac{1}{|t_{n-1}^{m}|}\beta \sum_{k\in t_{n-1}^{m}}q_{k}*q_{n} \end{array} \end{array}$$ (6)

Where $|t_{n-1}^{m}|$ represents the purchase count of *t*_*n*−1_-th transaction for user *m*. After sorting *pref* for all products, we recommend top-*K* products to customers.

### Optimization of the hybrid objective

When optimizing the hybrid objective, we find there is no closed-form solution for Eq (5). Here we introduce an alternative minimization algorithm to approximate the optimal results [24, 25]. The basic idea of this algorithm is to optimize the loss function with respect to one parameter, with all the other parameters fixed. The algorithm keeps iterating until convergence or the maximum of iterations. Specifically, we use *P*: |*U*| * *k*, *Q*: |*I*| * *k* stand for two low-rank matrix of customer set *U* and product set *I*.

**Algorithm 1** hybrid factoring sequential pattern and historical data

**Input:** :Input *R*, *W*, *m*, *n*, *k*, *α*, *β*, λ, *num*

**Output:** P,Q

i = 0

*P* ← *R*^m*k^, *Q* ← *R*^n*k^

**repeat**

*i* ← *i* + 1;

$P\leftarrow P\sqrt{\frac{\alpha RQ}{\alpha PQ^{T}Q+\lambda P}}$;

$Q\leftarrow Q\sqrt{\frac{\alpha R^{T}P+\beta W^{T}Q}{\alpha QP^{T}P+\beta QQ^{T}Q+\lambda Q}}$;

**until** converge or t≥ num

**return** *P*, *Q*;

First we fix parameter *Q*, and calculate value of *P* to minimize Eq (5). Let Λ be the Lagrange multiplier, then we get Lagrange function as follows:

$$\begin{array}{c} \begin{array}{c} \nabla (\alpha ∥\;R-PQ^{T}\;{∥}^{2}+\beta ∥\;W-QQ^{T}\;{∥}^{2}\\ +\lambda \left(∥\;P\;{∥}^{2}+∥\;Q\;{∥}^{2}\right)-tr\left(\Lambda P\right))=0 \end{array} \end{array}$$ (7)

the gradient is:

$$\begin{array}{c} \begin{array}{c} 2\alpha (R-PQ^{T})Q+2\lambda P-\Lambda =0 \end{array} \end{array}$$ (8)

With the given *KKT* complementary condition, we have the following equation:

$$\begin{array}{c} (2\alpha \left(R-PQ^{T}\right)Q+2\lambda P-\Lambda )P=0 \end{array}$$ (9)

Then we can obtain the gradient rule of *P*:

$$\begin{array}{c} P\leftarrow P\sqrt{\frac{\alpha RQ}{\alpha PQ^{T}Q+\lambda P}} \end{array}$$ (10)

The gradient rule of Q can be obtained in the similar way.

$$\begin{array}{c} Q\leftarrow Q\sqrt{\frac{\alpha R^{T}P+\beta W^{T}Q}{\alpha QP^{T}P+\beta QQ^{T}Q+\lambda Q}} \end{array}$$ (11)

The complete algorithm is shown in Algorithm 1.

### Complexity analysis

The learning algorithm has two parts: (1) Complexity of updating matrix P according Eq (10),which is *O*(|*U*| ⋅ |*I*| ⋅ *k*). (2) Complexity of learning matrix Q according Eq (11), which is *O*((|*U*|+ |*I*|) ⋅ |*I*| ⋅ *k*). Thus when using Algorithm 1 to learn our model, the total complexity is *O*((|*U*|+ |*I*|) ⋅ |*I*| ⋅ *k*). In the proposed approach, *k* is very small, so it has a very good practice performance, and we have found that it converges fast after only a few iterations.

## Experiment

### Data description

We conducted empirical experiments to evaluate the effectiveness of our proposed method on next-basket recommendation. The experiments were conducted on three real-world transactional datasets, including two retailer datasets: *BeiRen* and *Tafeng*, and one e-commerce dataset *TaoBao*.

The *BeiRen* dataset is collected by a large retail department store in China, recording purchase of products during the period from 2012 to 2013. The *Tafeng* datasetis released by RecSys, which covers products from food, office supplies to furniture. The *Taobao* dataset is an online e-commerce dataset released by Taobao, which records the online transactions in terms of brands.

First, we conduct pre-processes on these transactional datasets. For *BeiRen* dataset and *Tafeng* dataset, we filtered the products that were bought less than 10 times. For the *Taobao* dataset, which is quite small, we filtered the products that were bought less than 3 times, to obtain sufficient data for training and prediction. Details of the three datasets are shown on Table (1).

**Table 1:** Data set statistics.

id	name	# customers	# products	# transactions
1	*BeiRen*	13736	5920	242894
2	*Tafeng*	9238	7973	37269
3	*Taobao*	191	292	1805

We split all the datasets into training and testing sets, where the last transaction of each customer is taken as the test set, and all the previous transactions are taken as the training set.

### Baseline methods

To evaluate the recommendation performance of our model, we compare our model to several state-of-the-art next-basket recommendation methods, including the methods of item-centric and user-centric paradigms. We list all the baseline methods as follows:

- *TOP*: The most popular Top-*K* products are recommended.
- *NMF*: A state-of-the-art user-centric recommendation method based on collaborative filtering technique. Nonnegative Matrix Factorization is applied on customers’ historical purchase data. It can also be viewed as a sub-model of our method in which only the user-centric paradigm is adopted.
- *BPR*: A generic method for learning models of personalized ranking based on pairs of orders (i.e. the user-specific order of items) [22].
- *FPMC*: In *FPMC*, costumers’ sequential purchase behaviors are represented as a tensor and factorization is conducted to learn both low dimensional representations of customers and products [5].
- *FCSP*: A sub-model of our method in which only the item-centric recommendation paradigm is adopted. *FCSP* factorizes contiguous sequential patterns to learn low dimensional representations of products for next-basket prediction.
- *FSP*_1_: A sub-model of our method, which is an extension of *FCSP* by taking into account all the contiguous and non-contiguous sequential patterns. These two kinds of patterns are represented in a single matrix for learning and prediction.
- *FSP*_2_: A sub-model of our method, which is an extension of the *FCSP* method by taking into account all the contiguous and non-contiguous sequential patterns. The two kinds of patterns are combined linearly for learning and prediction.

For each method, we run 10 times, and take the average as the final result. Both CFSH and datasets are available at https://github.com/sgc1993/cfsh.git.

### Evaluation metric

We adopt *F*-measure as the evaluation measure for the Top-*K* Recommendation. *F*-measure is a weighted combination of precision and recall that produces scores of ranging from 0 to 1 and is accepted by many researchers as the metric for recommendation [5, 26–28]:

$$\begin{array}{c} F1-score=\frac{2\times Precision\times Recall}{Precision+Recall} \end{array}$$ (12)

### Comparison on different sequential models

**Fig 5:**
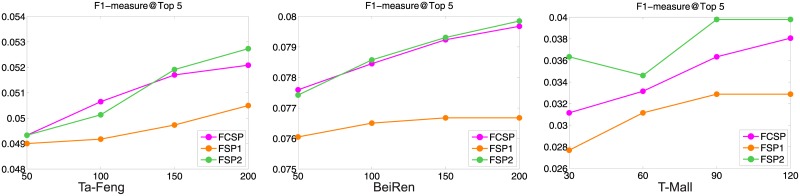
Comparison of factoring sequential patterns.

First, we evaluated the effectiveness of different item-centric models over sequential patterns, including *FCSP*, *FSP*_1_, and *FSP*_2_. The purpose is to test whether it is beneficial to involve long dependency between transactions. For the two extension models which mine patterns from non-consecutive transactions, we only consider the skip-1 transaction patterns. The results of the item-centric models over the three datasets are shown in Fig 5. From the results we can see that all the models perform similarly, which indicates that non-consecutive sequential patterns bring limited help for recommendation.

We analyzed the reasons. On one hand, the number of unique non-consecutive sequential patterns are much less than that of consecutive sequential patterns. If we took the weight into consideration, the difference could be even larger. Therefore, the learned representations of products will not be affected when the non-consecutive patterns were taken into account, because the optimization may still concentrate on the consecutive patterns. On the other hand, the non-consecutive sequential patterns contain more noise than consecutive ones. If we treat the non-consecutive patterns equivalently with the consecutive patterns, the noise may hurt the performance. By using the linear combination models with decayed weights on long dependent patterns, we can obtain similar or slightly better results.

Based on the above results, we use the item-centric model with only consecutive sequential patterns as our sub-model in the following comparison.

### Performance on next-basket prediction

In this section we compare our hybrid model to state-of-the-art methods in next-basket recommendation.

**Fig 6:**
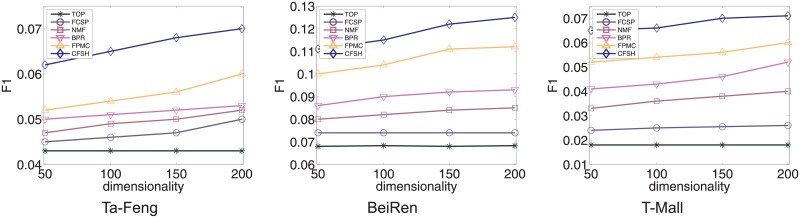
Comparison of our model *CFSH* to *TOP*,*FCSP*,*NMF*,*BPR*, and *FPMC* methods on three datasets.

Fig 6 shows the results on *Tafeng*, *Taobao* and *BeiRen* respectively. We can see the Top popular method perform worst on all three datasets. It indicates that the next-basket recommendation problem is not trivial. By only using the popularity of products, we cannot generate good performance on next-basket prediction. *NMF* perform better than *FCSP*, we assume that users’ long-term interests may be more important in predicting users’ purchase behaviours than users’ sequential patterns. Meanwhile, *BPR* can produce much better results than *NMF* and *FCSP* methods. This result show that in item recommendation domain directly optimizing for the task of personally ranking can perform better than traditional methods of recovering customer-product matrix. Moreover, by considering both item-centric and user-centric information, *FPMC* can obtain slightly better results over *BPR*. It demonstrates the benefit of combing the two kinds of information in recommendation. However, due to the sparseness of the tensor in *FPMC* and the difficulty in factorizing the tensor, the improvement is very small.

Comparing to other methods, our model outperforms all the state-of-the-art methods, with F1-score promoted at least 1.2% on *Tafeng* dataset, 1.1% on *BeiRen* dataset, 1.3% on *TaoBao* dataset respectively. The results demonstrate the effectiveness of combining two recommendation paradigms for next-basket prediction.

### Influenced of *α* on hybrid model

In this section we study the impact of parameter *alpha* in our hybrid model. Parameter *alpha* is a co-efficient which tunes the balance between item-centric and user-centric recommendation paradigms in our work.

When *alpha* approximates 1, the model turns into a pure user-centric model, which means that the customer is more likely to purchase products those similar customers have bought. When *alpha* approximates 0, the model becomes an item-centric model, and predict customers’ next purchase only relying on what he/she has bought in the latest transaction.

**Fig 7:**
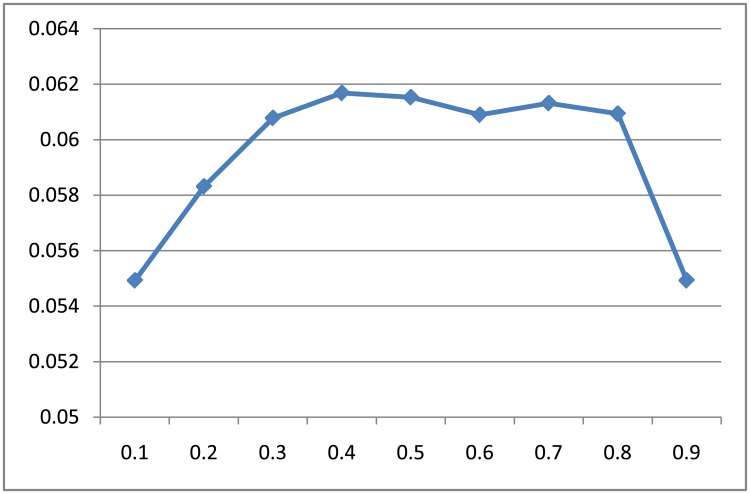
Performance of our model when adjusting alpha from 0.1 to 0.9 on *Tafeng* dataset with the dimension 50.

Here we show the results over different *α* on *Tafeng* dataset. Similar results can also be obtained from the other two datasets. We vary the value of *α* from 0.1 to 0.9 with the dimension of 50. Fig 7 show the performance results, where the F1-score is influenced by *α* especially on the two ends. When *α* takes the medial values, i.e. from 0.3 to 0.8, the performance is quite stable for our model.

## Conclusions

In this paper, we proposed a new hybrid recommendation method, namely *CFSH*, for next-basket prediction based on massive transactional data. The major purpose is to leverage the power of both item-centric and user-centric recommendation paradigms in capturing correlations between products and customers for better recommendation. This is achieved by factorizing customers’ sequential and historical purchase matrices simultaneously to learn customer and product representations better. Moreover, for the item-centric model, we propose to mine the sequential patterns from transactions in a global way to overcome the sparseness problem in sequential modeling. By conducting experiments on three real world purchase datasets, we demonstrated that our approach can produce significantly better prediction results than the state-of-the-art baseline methods.

In the future work, we would like to further analyze the correlations between products and costumers, so that we can better exploit this information and understand how these correlations affect each other. Moreover, we would like to integrate more context information into our model, e.g. time and location. Obviously, people’s shopping behavior may largely be affected by these factors. To present the next-basket recommendation at right time and right place would be very critical to task.
